# Recent Approaches for Manipulating Globin Gene Expression in Treating Hemoglobinopathies

**DOI:** 10.3389/fgeed.2021.618111

**Published:** 2021-08-02

**Authors:** Claudio Mussolino, John Strouboulis

**Affiliations:** ^1^ Institute for Transfusion Medicine and Gene Therapy, Medical Center–University of Freiburg, Freiburg, Germany; ^2^ Center for Chronic Immunodeficiency, Faculty of Medicine, University of Freiburg, Freiburg, Germany; ^3^ Laboratory of Molecular Erythropoiesis, Comprehensive Cancer Centre, School of Cancer and Pharmaceutical Sciences, King’s College London, London, United Kingdom

**Keywords:** hemoglobin, sickle cell disease, thalassemia, epigenome editing, targeted DNA methylation, targeted histone modification, designer epigenome modifiers, CRISPR-dCas9

## Abstract

Tissue oxygenation throughout life depends on the activity of hemoglobin (Hb) one of the hemeproteins that binds oxygen in the lungs and secures its delivery throughout the body. Hb is composed of four monomers encoded by eight different genes the expression of which is tightly regulated during development, resulting in the formation of distinct hemoglobin tetramers in each developmental stage. Mutations that alter hemoglobin structure or its regulated expression result in a large group of diseases typically referred to as hemoglobinopathies that are amongst the most common genetic defects worldwide. Unprecedented efforts in the last decades have partially unraveled the complex mechanisms that control globin gene expression throughout development. In addition, genome wide association studies have revealed protective genetic traits capable of ameliorating the clinical manifestations of severe hemoglobinopathies. This knowledge has fueled the exploration of innovative therapeutic approaches aimed at modifying the genome or the epigenome of the affected cells to either restore hemoglobin function or to mimic the effect of protective traits. Here we describe the key steps that control the switch in gene expression that concerns the different globin genes during development and highlight the latest efforts in altering globin regulation for therapeutic purposes.

## Introduction

**FIGURE 1:**
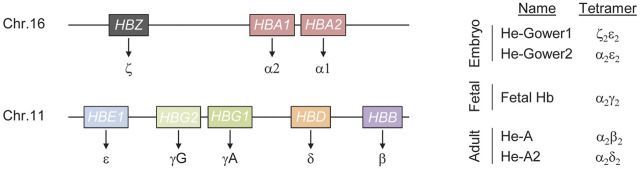
Schematics of human hemoglobin. Hemoglobin (He) is a tetrameric protein composed of two alpha and two non-alpha chains. Three genes (*HBS*, *HBA1* and *HBA2*) encode for the alpha chains while five genes (*HBE1*, *HBG1*, *HBG2*, *HBD* and *HBB*) result in non-alpha chains synthesis (left). Fine tuned regulation of these genes during development results in the formation of hemoglobin tetramers of different composition (right).

Hemoglobin (Hb) is the protein responsible for oxygen transport from lungs to all tissues and organs of the body. This is the most abundant protein of red blood cells (i.e. erythrocytes) and it is a tetramer, composed of two α and two β chains. Globin chains are encoded by eight different genes, three responsible for the α-like chains expression (ζ, α1 and α2) and five for the non-alpha chains (ε, γ1, γ2, δ, β; Figure 1). The expression of these genes is tightly regulated from embryogenesis to adulthood, thus resulting in dynamic alterations of the hemoglobin tetramer (Stamatoyannopoulos, 2005). Even though this is one of the most studied physiological processes worldwide, the mechanisms that fine-tune the delicate balance between the different globin chains are still elusive. Mutations in any of the aforementioned genes affect this delicate balance and, given the crucial role of hemoglobin, result in an heterogeneous group of hematopoietic defects named hemoglobinopathies which are typically divided into two major groups: 1) disorders of globin gene synthesis resulting in the reduction or loss of hemoglobin, typically referred to as *Thalassemias*, and 2) mutations leading to hemoglobin variants with structural abnormalities that result in a propensity to form protein aggregates (i.e. sickle syndromes), to precipitate or to have altered oxygen transportation properties (Trent, 2006). Since carriers of pathogenic globin variants approach 5–7% of the human population, hemoglobinopathies represent one of the most common human genetic disorder worldwide (Weatherall, 2008). This represents a serious concern for the quality of life of patients as well as for the health care systems facing the high-costs associated with its diagnostics and management. Treatment options for hemoglobinopathies are limited and typically depend on the severity of the disease. These might range from no treatment to regular transfusions and iron supplementation, or chelation depending on the clinical picture (Vinjamur et al., 2018). In the most severe forms, such as β-thalassemia major or sickle cell disease (SCD), hematopoietic stem cell transplantation is the only curative option but the risks of this procedure and the lack of suitable donors limit its applicability (Wienert et al., 2018). Therefore, strategies aiming at correcting the defect in the patients’ own cells prior to autologous transplantation are under scrutiny to treat patients with the most severe phenotypes. Gene addition strategies have shown some improvement in a broad range of β-thalassemia or SCD patients. To this end, autologous hematopoietic stem cells have been modified *ex vivo* prior to transplantation to express an exogenous copy of the β-globin gene to either complement the missing globin chain or to provide an anti-sickling effect, respectively (Urbinati et al., 2018). Interestingly, the evidence that residual HbF can ameliorate the condition of patients affected by β-thalassemia and sickle cell disease (SCD) (Paikari and Sheehan, 2018) and the study of a benign syndrome, the hereditary persistence of fetal hemoglobin (HPFH), have also been instrumental in developing innovative approaches to treat severe hemoglobinopathies. Different causes can lead to HPFH, such as 1) point mutations that either inhibit the binding of transcriptional repressors or create new binding sites for transcriptional activators in the γ-globin promoter, 2) large deletions that either remove inhibitory sequences or direct the LCR activity at the γ-globin promoter (Forget, 1998), or ultimately 3) the inheritance of quantitative trait loci variants capable of modulating HbF expression (Galarneau et al., 2010). In each case, the end result is elevated γ-globin levels in adults that contribute to alleviate disease symptoms in SCD patients (Sokolova et al., 2019). Strategies aimed at recreating the HPFH condition in SCD or β-thalassemic patients have gained momentum in the last decade and different genome editing technologies have been employed in this respect. Similarly, the reactivation of the endogenous dormant γ-globin gene has shown great potential ameliorating the β-thalassemia phenotype in patient derived stem cells (Makala et al., 2014). In this review we will describe the mechanisms that regulate globin gene expression throughout human life and illustrate how this knowledge might instruct novel therapeutic strategies to treat hemoglobinopathies in the future.

## Regulation of Hemoglobin Expression

Alteration of the hemoglobin tetramer composition during development results in hemoglobin molecules with different physical properties, capable of ensuring oxygenation to most tissues and organs throughout life. This plasticity, in response to the changing physiological needs for oxygen throughout development is ensured by a complex regulatory mechanism that defines the combination of genes expressed at different developmental stages. Regulation of α-like genes follows a relatively simple model. Five distal regulatory elements have been described in a region of about 50 kb upstream of the ζ-globin promoter (Flint et al., 2001). Of these elements, only two, typically referred to as Multispecies Conserved Sequence R1 and R2 (MCS-R1 and MCS-R2), are able to drive α-globin expression at high levels and behave as strong enhancers (Hay et al., 2016). The most widely accepted model of action for these enhancers is through the formation of a chromatin loop between the enhancers and the α-globin promoter (Vernimmen et al., 2009). Typically such elements are progressively bound by multiple transcription factors and cofactors that eventually establish chromatin modifications necessary to properly control target gene expression. In the case of α-globin, MCS-R1 and MCS-R2 are occupied by the P300 histone acetyltransferase that is capable of depositing lysine 27 acetylation on histone 3, a strong signal that identifies active chromatin (Higgs, 2013). In addition, binding of key transcription factors such as GATA-binding factor 1 (GATA1) and TAL bHLH transcription factor 1 (TAL1) further contribute to α-globin activation (Raffield et al., 2018). Besides a similar principle of regulation, the β-globin gene cluster expression is more complex. In the case of the β-like genes, high level expression is secured through the activity of the Locus Control Region (LCR), that includes five erythroid-specific DNaseI hypersensitive sites (DHS) located between ∼6 and ∼25 kb upstream of the first gene of the β-globin cluster (i.e. *HBE1*). These elements contain binding sites for transcription factors that play a crucial role during erythroid differentiation, such as GATA1 and TAL1 (Yun et al., 2014). The LCR activity is exerted through the formation of DNA loops that bring the enhancer in close proximity to the target β-like gene promoter (Forrester et al., 1986; Enver et al., 1990). This is favored and stabilized by the activity of LDB1 (Song et al., 2010). The long-range interaction occurring between the LCR and the promoters of the β-like genes are dynamic during development and determine which gene is activated (Tolhuis et al., 2002; Vernimmen et al., 2007). During fetal development, the LCR associates preferentially with the γ-globin promoter resulting in high levels of *HBG* gene expression while after birth, the LCR engages with the β-globin promoter, resulting in the overexpression of the distal *HBB* gene. A second mechanism that contributes to shaping the gene expression profile of β-like globin genes is the direct silencing of the embryonic and fetal globin chains (Behringer et al., 1990). As a result of the fine-tuned regulation of the α- and β-like genes, hemoglobin molecules of different composition form during development (Figure 1). In the embryo, expression of the three α-globin genes (*HBZ*, *HBA1*, *HBA2*) and of the β-like globin gene *HBE1* leads to the formation of He-Gower1 (ζ_2_ε_2_) and He-Gower2 (α_2_ε_2_). After the first trimester, *HBZ* is silenced and *HBA* remains the only α-chain expressing gene active throughout life (Mettananda et al., 2016). After the first trimester also *HBE* is silenced while the genes encoding mainly for γ-globin and to a lesser extent β-globin, are upregulated. At this stage, the predominant γ-globin pairs with α-globin forming the so-called fetal hemoglobin (HbF). Perinatally, γ-globin gene expression declines gradually and β-globin remains the predominant non-alpha chain expressed throughout life forming together with α-globin the adult hemoglobin (HbA) (Stamatoyannopoulos, 2005; Manning et al., 2007). However, low γ-globin expression persists during adulthood giving rise to a small population of cells containing HbF and typically referred to as F cells (Thein and Menzel, 2009). Around birth also the gene expressing of another non-alpha chain, the δ-globin gene, is upregulated and its low expression level persists for the entire life. Pairing between δ- and α-globins results in the formation of hemoglobin A2 (HbA2) (Wienert et al., 2018). This variant typically accounts for about 2.5% of total Hb in adult healthy individuals. However it tends to be elevated in thalassemic individuals highlighting its importance in screening programs (Giambona et al., 2009).

The γ-to-β globin switch is the subject of extensive studies as it plays a major role in the disease manifestation of β-hemoglobinopathies. Increased fetal hemoglobin levels can indeed improve the pathology of patients suffering from hemoglobinopathies of different etiology. Therefore, understanding the mechanism behind the switch is crucial to develop novel therapeutic strategies to combat this multifaceted disease group. To date, multiple factors have been involved in this complex mechanism. BCL11A is a dominant regulator responsible for the silencing of γ-globin in humans (Sankaran et al., 2008). The corresponding *BCL11A* gene is highly expressed in adult erythroid cells where γ-globin levels are low and vice versa in fetal erythroid cells. While haploinsufficiency of BCL11A consistently associates with increased HbF levels, it is also responsible for neurocognitive defects (Basak et al., 2015), highlighting the important role this protein has in different developmental processes. The mechanism of action of this factor in controlling γ-globin gene expression has been studied in detail, even though aspects of its function remain unclear. Additionally, BCL11A has been also shown to directly interact with a major repressor complex, the nucleosome remodeling and deacetylase (NuRD). This complex includes histone deacetylases which are critical for the silencing of γ-globin gene (Cao et al., 2004) suggesting a role for BCL11A in mediating NuRD localization to γ-globin promoter. Another key factor implicated in γ-globin silencing is the Leukemia/lymphoma-related factor (LRF). Both loss of this factor and mutations in its binding site in the γ-globin promoter result in γ-globin expression (Masuda et al., 2016; Weber et al., 2020). Independently from BCL11A, LRF is also able to interact with multiple epigenetic repressors and with the NuRD complex mediating the formation of heterochromatin at the γ-globin promoter (Masuda et al., 2016). Therefore, multiple pathways contribute to the deposition of crucial repressor complexes capable of keeping the promoter of the γ-globin gene in a closed chromatin configuration. The major contribution of the epigenome to the γ-to-β globin switch is further confirmed by the activity of another multi-factor complex that has been associated with both ε- and γ-globin silencing, namely, the direct repeat erythroid-definitive (DRED) complex. This is comprised of mainly the nuclear receptors TR2/TR4 interacting with multiple other protein partners such as NuRD, histone demethylases and DNA methylase. Other crucial upstream regulators capable of controlling the expression of multiple downstream effectors have also been identified and studied. For example, silencing of MYB has been directly linked to the downregulation of multiple key players in *HBG* silencing such as BCL11A and TR2/TR4 with consequent elevation in γ-globin expression (Roosjen et al., 2014). Interestingly, MYB also directly controls the expression levels of KLF1, a potent activator of BCL11A suggesting that these master regulators (Borg et al., 2010) of globin genes are tightly interconnected and their interplay essential to fine-tune globin gene expression. Continuing efforts to elucidate this complex network of globin gene regulation is certainly necessary to understand many, still elusive, aspects and to shed light on the entirety of this convoluted process.

## Altering Globin Gene Regulation for Therapy

**FIGURE 2:**
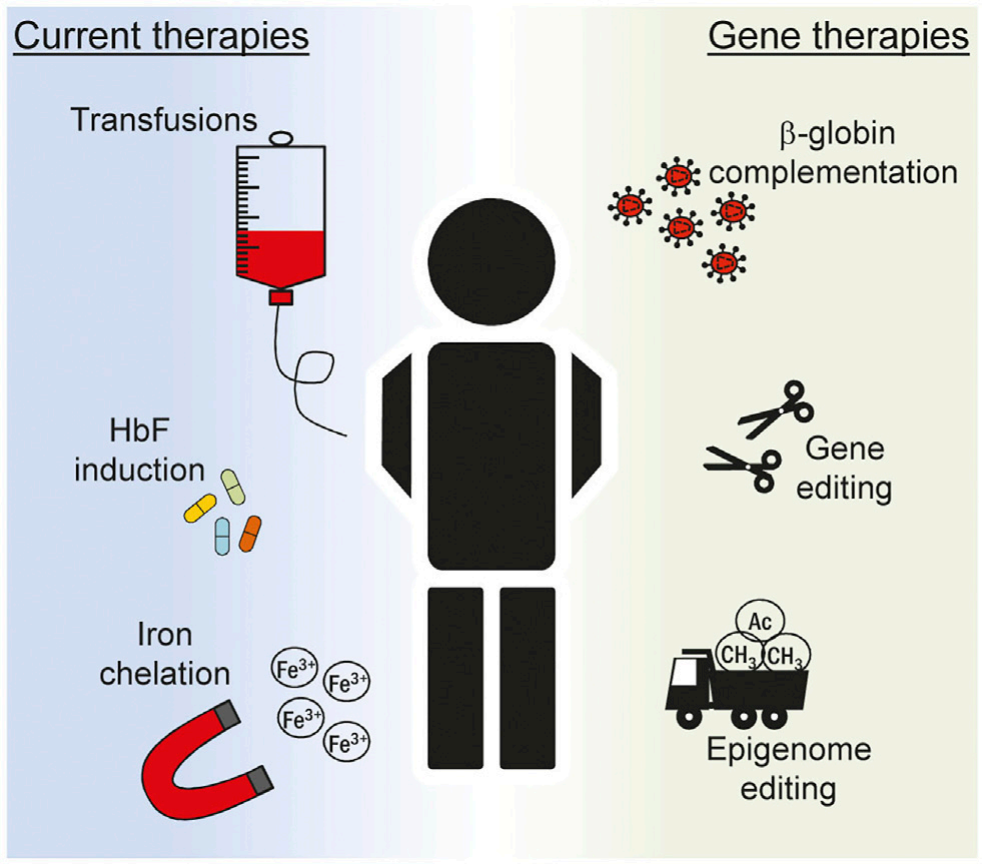
Overview of current treatments for hemoglobinopathies. Multiple strategies are devised to ameliorate the clinical manifestation of hemoglobinopathies in patients. Current treatments (left) include recurrent blood transfusions, pharmacologic induction of fetal hemoglobin (HbF) expression and removal of iron excess. The knowledge acquired on the complex mechanism that regulates globin expression has boosted in recent years the inception of novel gene therapeutics (right). *Ex vivo* editing of autologous stem cells can be explored to correct the genetic defect and restore normal hematopoiesis. In this context lentiviral vectors expressing the β-globin gene can be used to correct the phenotype of patients suffering from the most severe hemoglobinopathies, such as β-thalassemia and sickle cell disease. More recently, genome and epigenome editing technologies are explored to alter globin gene regulation in order to reactivate HbF to mimic the protective effect of genetic traits typical of hereditary persistence of fetal hemoglobin (HPFH) condition.

The last decades has witnessed a tremendous effort in understanding the pathways controlling hemoglobin expression and in dissecting the mechanisms behind its failure leading to hemoglobinopathies of different gravity. Regular transfusions and iron chelation are currently among the gold standard treatment options for β-hemoglobinopathies (Figure 2) (Fibach and Rachmilewitz, 2017). The evidence that residual HbF can ameliorate the condition of patients affected by β-thalassemia and sickle cell disease (SCD) (Paikari and Sheehan, 2018) prompted researchers to better understand the regulation of γ-globin gene in an attempt to reactivate its expression for the patients’ benefit. Pharmacologic induction of HbF is presently achieved via administration of Hydroxyurea (HU). This compound was approved by the U.S. Food and Drug Administration (FDA) in the late 90s and by the European Medicines Agency (EMA) in early 2000 and since then remains the only drug available for the induction of HbF. Patients typically take this medication orally every day and this regimen typically results in reduced transfusions (Charache et al., 1995) and increased life expectancy (Voskaridou et al., 2010). However, compliance to therapy has been often hindered by side-effects, such as male impotency and leukemogenic potential, as have been reported in some cases (Segal et al., 2008; DeBaun, 2014). Importantly, the mechanism of action of HU has not been clarified in full thus far. Several pathways have been proposed including epigenetic modifications (Walker et al., 2011), direct alteration of major intracellular pathway (Park et al., 2001) and post-transcriptional regulation achieved through changes in key miRNA expression (Mnika et al., 2019). Given the uncertainty around the exact mechanism through which HU induces HbF, a thorough characterization of its molecular mode of actions is surely paramount to improve its safety and ameliorate therapeutic regimens.

The importance of epigenetic mechanisms in the regulation of globin gene expression has fueled the search for novel compounds capable of altering the epigenome for therapeutic purposes. Epigenetic regulation can act through multiple mechanisms that eventually lead to chromatin alterations that either permit or inhibit transcription (Mussolino, 2018). Both DNA methylation, nucleosome remodelling and histone modifications, such as acetylation and methylation, play crucial roles in regulating the expression of γ-globin genes (Mabaera et al., 2007; Kiefer et al., 2008). Furthermore, the evidence that all transcriptional repressors of adult γ-globin expression interact with and recruit epigenetic enzymatic activities to the γ-globin promoters (Ginder, 2015; Yu et al., 2020), has opened up a window of opportunity for blocking these activities in reactivating γ-globin as an effective approach for treating hemoglobinopathies. For example, it has been known for some time that treatment with 5-azacytidine results in degradation of the DNMT1 DNA methyltransferase and induction of γ-globin expression in adult erythropoiesis [reviewed in (Saunthararajah et al., 2004)]. Recent clinical trials using decitabine (a less toxic 5-azacytidine derivative) combined with tetrahydrouridine (THU, a decitabine stabilizer), have shown significant promise in treating sickle cell disease (Molokie et al., 2017). In agreement with these findings, DNMT1 was shown to interact with key γ-globin transcriptional repressors, including BCL11A, DRED and GATA1 (Cui et al., 2011; Xu et al., 2013; Papageorgiou et al., 2016). In addition, recent work identified a DNMT1 germline missense mutation giving rise to HPFH (Gong et al., 2021), thus providing direct evidence for DNMT1 as a key γ-globin epigenetic co-repressor and further validating it as a therapeutic target for hemoglobinopathies (Saunthararajah, 2021).

Induction of γ-globin expression through histone deacetylase (HDAC) inhibition by butyrate, is another example where treatment preceded the detailed molecular characterization of HDAC involvement in γ-globin repression (Ikuta et al., 1998). Subsequent work showed HDAC1 and HDAC2 to be members of NuRD and of the LSD1/CoREST complex, both implicated in γ-globin repression through interactions with BCL11A (Xu et al., 2013), DRED (Cui et al., 2011) and LRF [NuRD only (Masuda et al., 2016)]. Several HDAC inhibitors were subsequently developed and tested (Bradner et al., 2010), with *vorinostat* recently approved by the FDA for γ-globin reactivation in the clinic (Okam et al., 2015) and additional modulators currently being tested in early-stage trials (Yu et al., 2020). However, the therapeutic use of HDAC inhibitors suffers from toxicity due to non-specific effects and from risks associated with an incomplete understanding of HDAC1, HDAC2 and HDAC3 distinct and overlapping functions in erythropoiesis, which may be detrimentally affected by their indiscriminate inhibition [recently reviewed in (Wang et al., 2021)].

The histone demethylase LSD1 has also emerged as a key epigenetic repressor of γ-globin expression. LSD1 is a member of the CoREST complex shown to interact with BCL11A and DRED (Cui et al., 2011; Masuda et al., 2016). Inhibition of LSD1 using tranylcypromine or RN-1 was shown to result in γ-globin reactivation in human cells, mice and baboons and to alleviate anemia in a transgenic sickle cell mouse model [recently reviewed in (Yu et al., 2020)]. Other epigenetic enzymatic activities implicated in γ-globin repression that have potential to serve as druggable targets in hemoglobinopathies include the EHMT1/2 histone methyltransferase heterodimer and the PRMT5 and PRMT1 protein arginine methyltransferases. The EHMT1/2 heterodimer has been shown to act as a repressor of embryonic and fetal globin genes and as an activator of adult globin genes (Chaturvedi et al., 2009). Inhibition of EHMT1/2 in human cells with the small molecule UNC0638 led to γ-globin reactivation with no overt effects on erythropoiesis (Renneville et al., 2015), thus making it a promising target for therapeutic applications. PRMT5 has been implicated in γ-globin repression through its participation in a multi-protein complex that also involves NuRD and the Suv4-20h1 histone methyltransferase (Rank et al., 2010). PRMT1 was shown to be involved in γ-globin repression through its recruitment to chromatin by CHTOP (van Dijk et al., 2010). Of note, a number of PRMT small molecule inhibitors specifically targeting PRMT5 and PRMT1 have been developed and are now entering clinical trials, but not yet for treating hemoglobinopathies (Li et al., 2019).

Another approach for targeting epigenetic repressors of γ-globin repression is through their specific protein degradation. An early example is the use of decitabine to induce DNMT1 degradation (see above). Another example involves the recently described deubiquitinase BRCA1-associated protein-1 (BAP-1) which was shown to protect the DRED scaffolding protein NCoR1 from ubiquitin-mediated degradation. Importantly, knocking down BAP-1 led to NCoR1 ubiquitination and degradation, resulting in robust γ-globin reactivation and HbF expression (Yu et al., 2018). Lastly, the development of so-called Proteolysis Targeting Chimeras (PROTACs) for the degradation of targeted proteins through the specific recruitment of E3 ubiquitin ligase, has been proposed as an alternative approach for targeting not only epigenetic co-repressors, but also transcriptional repressors such as BCL11A (J Verheul et al., 2020), but has yet to be demonstrated in practice in the reactivation of γ-globin expression.

From the work outlined above, the NuRD chromatin remodelling and histone deacetylase complex has emerged as a near-universal co-factor to all transcriptional repressors involved in globin gene switching (Lee et al., 2017). As a result, NuRD has become the focus of intense investigation in dissecting it as a possible target for HbF reactivation in treating hemoglobinopathies. Recent work by Bauer and others employed a novel CRISPS/Cas9 dense *in situ* mutagenesis to identify the domains of NuRD protein sub-units that can be targeted to increase HbF levels without compromising cell viability or erythroid differentiation (Sher et al., 2019). This led to the identification of the C-terminal CHDCT2 domain in the CHD4 protein (a core component of NuRD) which is responsible for the recruitment of CHD4 to NuRD through binding of the GATAD2A protein, also a NuRD core component. Importantly, use of a GATAD2A peptide sequestered CHD4 away from the NuRD complex by disrupting the endogenous CHDCT2/GATAD2A protein interactions, resulting in high levels of HbF expression with minimal toxicity. Further work targeting the NuRD complex carried out in parallel by the Bauer and Blobel groups, led to the astounding discovery of ZNF410 as a transcription factor that is solely dedicated to activating CHD4 expression in erythroid cells (Lan et al., 2021; Vinjamur et al., 2021). Targeting ZNF410 in erythroid cells led to CHD4 downregulation and HbF induction, without compromising erythroid cell differentiation, thus validating ZNF410 as a promising potential target for treating hemoglobinopathies.

Taken together, targeting the epigenetic and chromatin remodelling co-factors implicated in γ-globin repression is proving a very promising approach for HbF reactivation that is beginning to find its way into the clinic, but challenges remain as to potential toxicities and detrimental effects on erythropoiesis. The recent application of high throughput CRISPR/Cas9-based functional screens is paving the way for the identification of targetable protein interaction domains that disrupt epigenetic co-factor functions in driving high HbF levels without compromising cell viability and differentiation.

## Gene Therapy

Gene therapy offers the opportunity to modify the cellular genome with different degrees of precision both at the genome and at the epigenome level (Figure 2). Randomly integrating viral vectors expressing β-globin are currently exploited in multiple trials to correct the phenotype of β-thalassemic and SCD patients (Cavazzana-Calvo et al., 2010; Ikawa et al., 2019). With the inception of designer nucleases (DNs), virtually any site in the human genome can be targeted with nucleotide precision (Carroll, 2014). The resulting DN-induced double strand break is typically repaired via the non-homologous end-joining DNA repair pathway producing small insertion and deletion (indel) mutations at the target site (Carusillo and Mussolino, 2020). Targeting DNs to known repressive sequences in the γ-globin promoter has been attempted in order to induce indel formation thus abolishing the binding of γ-globin repressors. In a recent report, this strategy resulted in about 50% of circulated red blood cells (RBC) expressing HbF seventeen weeks after transplantation of edited hematopoietic stem cells (HSC) in a mouse model (Métais et al., 2019). Similarly, the simultaneous use of multiple DNs has been envisioned to recreate deletions in the β-globin locus resulting in a HPFH-like condition with HbF levels approaching 60% of total hemoglobin in RBCs derived from edited HSCs (Antoniani et al., 2018). Since indel formation is random and the high frequency of DSBs might be deleterious (Conti and Di Micco, 2018), alternative strategies have been envisioned that do not rely on genomic breakage. Base editors offer the possibility to induce DNA base pair conversion without inducing a DSB. Cytosine base editors have been developed to convert C:G to T:A base pair (Komor et al., 2016). In the context of γ-globin promoter, they have been recently used to create specific nucleotide substitutions to abolish the binding of the BCL11A repressor (Wang et al., 2020), reaching also in this case clinically relevant levels of HbF after differentiation of edited HSCs. Similar strategies have been explored to create novel binding sites for known globin activators. Again, the knowledge acquired from HPFH studies revealed that a mutation in the γ-globin promoter, namely the −198 T to C also called British variant, results in elevated fetal globin levels. Recent studies have demonstrated that this effect is consequent to the creation of a novel binding site for the erythroid transcriptional activator KLF1 (Wienert et al., 2017). This has fueled the use of base editors to specifically recreate the −198 T to C mutation in cell lines (Gaudelli et al., 2017). The DNA targeting platforms used in the context of genome editing can also be employed to achieve targeted changes of the epigenome. The epigenetic drugs described in the previous section are non-specific and their global effect poses serious safety concerns. Engineered epigenetic effectors capable of targeted epigenome editing represent a solution to this problem and are currently under scrutiny in different pathological situations (Gjaltema and Rots, 2020). Epigenome editors based on the CRISPR-Cas9 system have been used to directly reactivate γ-globin expression. In this case, commonly used viral activation domains such as the VP64 or the catalytic domain of the acetyltransferase p300 have been fused to a catalytically inactive Cas9 (dCas9) to directly reactivate γ-globin transcription in cell line (Hilton et al., 2015). Similarly, fusion of the ten-eleven translocation (TET) 1 demethylase to a TALE DNA binding domain targeted to the β-globin promoter efficiently reactivated the *HBB* gene through targeted DNA demethylation in an erythroid cell line (Maeder et al., 2013). We have developed designer epigenome modifiers (DEMs) which are capable of depositing both DNA and histone methylation, such as H3K9me3, at defined target sites (Mlambo et al., 2018). Since epigenetic regulation at the globin loci is complex, it is reasonable to think that the fine-tuned expression of the different genes involved requires interplay between DNA methylation and histone modifications. Therefore, the technological advancement offered by DEMs might be of great value as they combine in a single molecule the ability to alter the epigenome both at DNA and histone levels. While we have shown that these effectors can be efficiently used to silence the expression of genes that promote HIV infection (Mlambo et al., 2018) or T cells exhaustion (Roman Azcona et al., 2020), it will be interesting to prove their potential in silencing repressors of γ-globin gene, such as BCL11A, to promote *HBG* reactivation. Even though an approach that results in broad BCLA11A suppression is likely detrimental for the crucial role of this transcription factor in non-erythroid cells (Luc et al., 2016), the recent evidence that this gene can be ablated in an erythroid-specific fashion (Brendel et al., 2016) provides a new opportunity to be explored in the epigenome editing field. These studies clearly show the capability offered by these novel technologies for the treatment of complex human disorders and more efforts are certainly necessary to establish the therapeutic potential of such innovative therapeutics.

## Conclusion

The many studies conducted to dissect the genetic and epigenetic regulation of the globin genes expression has highlighted multiple potential therapeutic strategies to tackle β-hemoglobinopathies. This knowledge, combined with the inception of targeted genome and epigenome editing platforms offers the unique opportunity to explore exciting avenues for developing innovative medicines. Proper evaluation of the risks associated with the use of these pioneering technologies in transplantable stem cells will certainly propel their exploitation in clinical studies to explore their potential for patients’ benefit. Considering the ongoing clinical efforts [recently reviewed in (Ikawa et al., 2019; Magrin et al., 2019)], this will eventually contribute to the dawn of a novel class of targeted therapeutics to treat disorders of the blood.
